# Characterization of Fungal Colonization of Indwelling Esophagostomy Tubes

**DOI:** 10.1155/2019/8153468

**Published:** 2019-06-25

**Authors:** Shelly J. Olin, David A. Bemis, John R. Dunlap, Jacqueline C. Whittemore

**Affiliations:** ^1^Department of Small Animal Clinical Sciences, University of Tennessee, College of Veterinary Medicine, 2407 River Drive, Knoxville, TN 37996, USA; ^2^Department of Biomedical and Diagnostic Sciences, University of Tennessee, College of Veterinary Medicine, 2407 River Drive, Knoxville, TN 37996, USA; ^3^Joint Institute for Advanced Materials, University of Tennessee, 2641 Osprey Vista Way, Knoxville, TN 37996, USA

## Abstract

Fungal colonization of feeding tubes occurs rapidly in people, resulting in decreased structural integrity and complications such as luminal obstruction and tube failure. Esophagostomy tubes (E-tubes) are commonly used in dogs and cats for enteral support, but data are lacking regarding colonizing fungi and the impact of colonization on tube integrity. In this study, esophagostomy tubes were collected in lieu of disposal from dogs and cats undergoing feeding tube exchange. Fungi were isolated with culture and identified using morphological characteristics. Scanning electron microscopy was used to evaluate the surface characteristics of the tubes. Two silicone and one polyurethane E-tube were evaluated. Fungi associated with the normal microbiota, including* Candida *sp. and* Penicillium* sp., as well as environmental fungi were identified. This case series represents the first documentation of fungal colonization of silicone and polyurethane E-tubes in dogs and cats. Additionally, this is the first report to document degenerative changes in a silicone E-tube.

## 1. Introduction

Fungal colonization of feeding tubes occurs rapidly in people and is associated with decreased structural integrity, luminal obstruction, and tube failure [1–5]. Fungal colonization is characterized by dense, creamy, or darkly pigmented growth on the luminal tube surface [3, 4]. Certain fungi use alkanes, a hydrocarbon plasticizer-stabilizer in silicone and polyurethane tube walls, as a carbon source [1, 3, 5, 6]. Additionally, some fungi, such as* Candida albicans*, produce hydrolytic enzymes that degrade polyurethane in feeding tubes [5]. The net result is the development of porous holes and crevices in the tube wall [5, 7]. The most prevalent fungal colonizer of human gastrotomy tubes (G-tubes) is* Candida *sp., but many others are reported [2–5].

Esophagostomy tubes (E-tubes) are commonly used in dogs and cats for enteral support [8]. Although we have observed creamy and darkly pigmented growth, consistent with fungal colonization, on the luminal surface of long-term indwelling E-tubes in dogs and cats (personal observation, 2018), data are lacking regarding colonizing fungi and the impact of colonization on tube integrity. The objectives of this case series were to identify the fungal organism(s) colonizing E-tubes and to describe associated morphologic changes to the luminal surface of colonized tubes.

## 2. Case Presentation

Esophagostomy tubes were collected in lieu of disposal between July 1, 2017, and June 30, 2018, from dogs and cats undergoing feeding tube exchange at the University of Tennessee, Veterinary Medical Center. Tubes were selected for inclusion if there was visible creamy or pigmented colonization on the luminal tube surface. The protocol for tube exchange at this institution was to advance a sterile hydrophilic guide-wire through the indwelling tube into the distal esophagus or stomach with the patient lightly sedated. Once sufficient wire was advanced to avoid risk of accidental dislodgement, the anchoring suture was cut and the tube removed over the wire. A new sterile tube then was fed over the guide-wire and sutured in place after radiographic confirmation of tube insertion depth.

Once a tube was removed from the animal, it was examined grossly and photographed. The external surfaces of evaluated feeding tubes were wiped with 70% isopropyl alcohol (Medline, Mundelein, IL) and allowed to air-dry at room temperature. Tubes were transected longitudinally with sterile technique. Representative creamy or darkly pigmented areas were collected using a sterile scalpel blade. 

*Fungal Isolation and Identification*. Lactophenol aniline blue stain (Remel, Lenexa, KS) and Gram stain (Remel, Lenexa, KS) were used. Slides were examined under 4x and 10x power using light microscopy. Morphologic characteristics of identified fungi were recorded. Cultures were performed in accordance with established laboratory protocol. Briefly, a sterile loop calibrated to hold a volume of 0.001 mL was used to inoculate the sample onto 2 plates: Inhibitory Mold Agar (Remel, Lenexa, KS) and Sabouraud Dextrose Brain/Heart Infusion Agar (Remel, Lenexa, KS). The cultures were incubated at 30°C for 6 weeks. Fungal identification was confirmed by morphological analysis. 

*Scanning Electron Microscopy*. Representative sections of the tube were cut into 4-cm long equal parts and fixed in Carson's buffer. After primary fixation, samples were washed in distilled water for 30 minutes and then postfixed in 2% aqueous osmium tetroxide for 60 minutes. Samples were washed in three exchanges of water over 30 minutes before being dehydrated in a graded ethanol series and finally air-dried for at least 24 hours. Dried samples were mounted on sample holders and sputtered with gold (SPI Sputter Coater, SPI, Inc.) before imaging in a Zeiss Auriga Dual Beam SEM (Zeiss, Oberkochen, Germany).

### 2.1. Case 1

**Figure 1:**
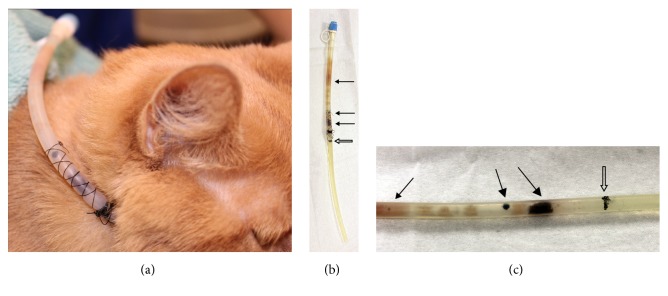
A silicon esophagostomy tube exposed for 77 days in a cat (case 1). (a) The esophagostomy tube prior to removal. Note the multifocal darkly pigmented plaques, primarily within the area of the suture finger trap at the site of tube insertion. (b) The esophagostomy tube after removal. Solid black arrows highlight darkly pigmented plaques, while thinner, lighter colored plaques extend up the insertion tube. The hollow arrow indicates a marker line at the site of interface of the tube and stoma. Note the lack of plaques below the interface line. (c) Close-up photo of the esophagostomy tube after removal of the suture finger trap. Solid black arrows highlight the most prominent plaques. The hollow arrow indicates a marker line at the site of insertion into the stoma.

A 2-year female spayed domestic shorthair undergoing treatment for severe chronic rhinitis and bacterial pneumonia had a silicone E-tube (Jorgensen Laboratories, Inc., Loveland, CO) exchanged because of darkly pigmented growth on the tube's luminal surface of 3-4 day duration (Figures 1(a)-1(c)). At the time of the exchange, the cat had an E-tube in place for a total of 154 days, with the most recent tube exchange being 77 days earlier because of accidental dislodgement.

**Figure 2:**
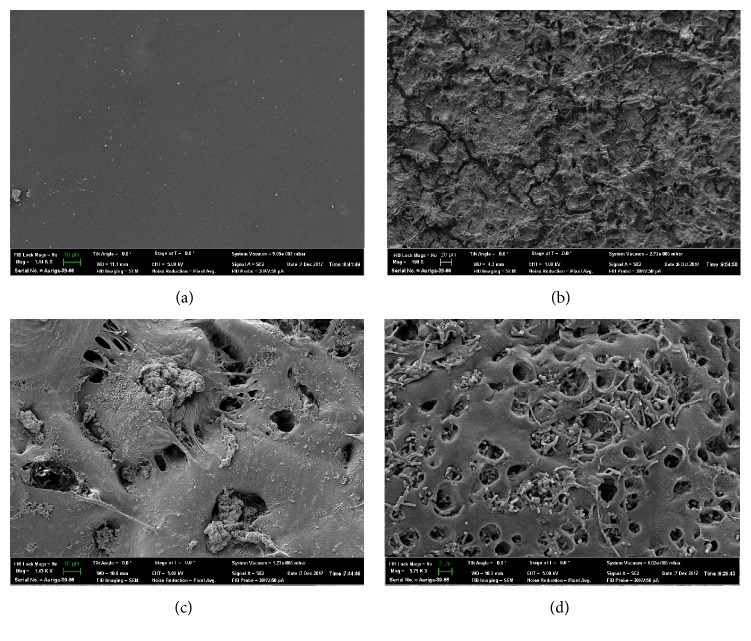
Scanning electron microscopy (SEM) images of silicone esophagostomy tubes. (a) The luminal surface of a reference tube. The surface is mostly regular with mild imperfections. The scale bar is equal to 10 *μ*m and the original magnification is 1.44K. Photo credit: John Dunlap. (b) Thick biofilm with crevices covering the luminal surface of a tube exposed for 77 days in a cat (case 1). Dense mats of fungal mycelia are visible. The scale bar is equal to 20 *μ*m and the original magnification is 450x. Photo credit: John Dunlap. (c) Biofilm and multiple large pores in the luminal wall of the intraesophageal portion of a tube exposed for 246 days in a dog (case 2). The scale bar is equal to 10 *μ*m and the original magnification is 1.43K. Photo credit: John Dunlap. (d) Mixed biofilm with bacteria and yeast, as well as a multitude of pores, in the luminal wall of the extraesophageal portion of a tube exposed for 246 days in a dog (case 2). The scale bar is equal to 2 *μ*m and the original magnification is 5.75K. Photo credit: John Dunlap.

Multifocal dense, brown, pigmented growth was visible near the site of tube insertion into the body. The growth would not scrape off the surface of the tube, so the superficial layer of the tube was scraped to collect a sample for culture and identification. Multicell macroconidia of a melanin producing filamentous fungus were identified with light microscopy.* Curvularia* sp. was identified on fungal culture. Scanning electron microscopy findings included a thick biofilm with matts of fungal mycelia and curved macroconidia characteristic of* Curvularia* spp. The biofilm was too dense to allow evaluation of the underlying tube surface (Figures 2(a) and 2(b)).

### 2.2. Case 2

A 15-year female spayed Toy Poodle with inflammatory bowel disease had a silicone E-tube (Jorgensen Laboratories, Inc., Loveland, CO) exchanged because of darkly pigmented growth noted near the site of tube insertion and an odor of yeast for approximately 5-day duration. At the time exchange, the dog had an E-tube in place for a total of 1,142 days, with the most recent tube exchange being 246 days earlier because of gagging and retching. Preceding that exchange, the dog had undergone two exchanges in the year preceding the most recent tube exchange because of darkly discolored growth on the tube. At that time, it was discovered that the owners never disinfected or replaced syringes used for feeding; the frequency of discolored growth in the tube decreased substantially after changing management practices.

The stoma site was malodorous and exudative. The portion of the tube outside of the body had a diffuse brown discoloration. After tube removal, dark luminal discoloration was identified extending 1.5 cm beyond the insertion site. The external surface of the portion of the tube withdrawn from the esophagus also was diffusely covered with a white exudate and smelled of yeast. Separate samples, thus, were collected from the luminal and extraluminal portions of the tube. Growth from the luminal surface of the tube was flaky and easy to remove from the tube surface. Light microscopy findings were consistent with yeast.* Penicillium* sp.,* Candida* sp. (not* C. albicans*), and a* Trichosporon*-like yeast were identified on fungal culture. Growth on the extraluminal aspect of the tube was more adhered. Culture was consistent with* Candida* sp. Scanning electron microscopy findings included a thick biofilm and numerous, variably sized pores within the tube wall, consistent with tube degradation (Figures 2(c) and 2(d)). Stoma-site cellulitis resolved after tube exchange.

### 2.3. Case 3

A 13-year male neutered Chihuahua with copper storage hepatopathy had a polyurethane E-tube (MILA International, Florence, KY) exchanged because of visible cream-colored blotches of unknown duration in the tube. At the time of the exchange, the dog had an E-tube in place for a total of 524 days, with E-tube exchange last performed 204 days earlier because of accidental dislodgement.

**Figure 3:**
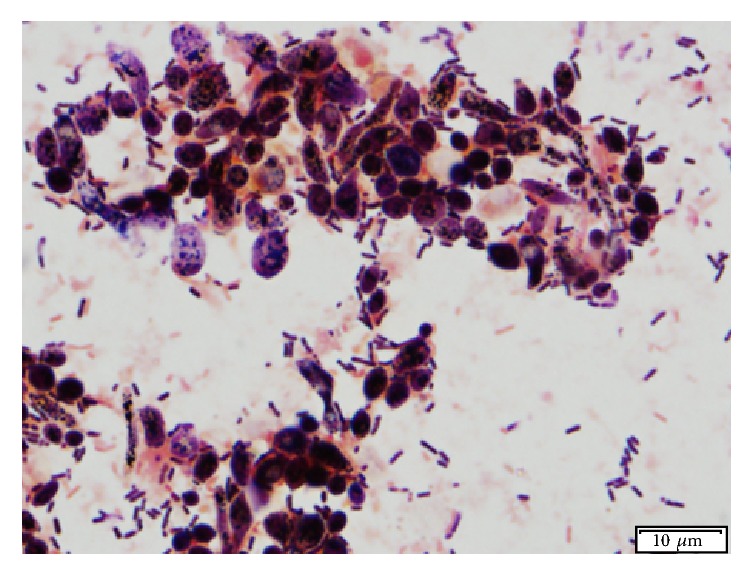
1000x magnification of mixed bacteria and budding yeast like cells on Gram stain from a dog (case 3). Photo credit: Bente Flatland.

**Figure 4:**
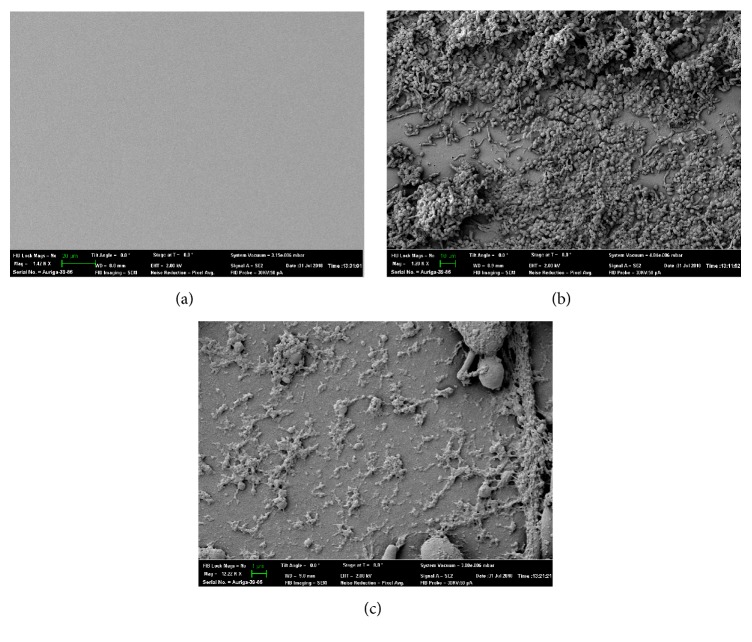
Scanning electron microscopy (SEM) images of polyurethane esophagostomy tubes. (a) The luminal surface of an unused polyurethane tube. The scale bar is equal to 20 *μ*m and the original magnification is 1.42K. Photo credit: John Dunlap. (b) Thick bacterial and yeast-containing biofilm on the luminal surface of a tube exposed for 204 days in a dog (case 3). The visible tube surface has no evidence of pores or cracks. The scale bar is equal to 10 *μ*m and the original magnification is 1.28K. Photo credit: John Dunlap. (c) Thin biofilm on the smooth luminal surface of a tube exposed for 204 days in a dog (case 3). The scale bar is equal to 1 *μ*m and the original magnification is 12.22K. Photo credit: John Dunlap.

Diffuse whitish colored growth covered the majority of the luminal surface of the portion of the tube that had been outside the body. Lighter growth was noted on the luminal surface of the section of the tube that had been intraesophageal. Small, round to ovoid budding yeast with occasional pseudohyphae were observed by light microscopy (Figure 3).* Candida albicans* was identified on fungal culture. A thick biofilm of bacteria and yeast was evident on SEM, but the visible areas of the luminal E-tube surface appeared smooth; pores and crevices were not noted (Figure 4).

## 3. Discussion

This case series represents the first documentation of fungal colonization of silicone and polyurethane E-tubes in dogs and cats. Additionally, this is the first report of degenerative changes in a silicone E-tube. A complex microenvironment can develop in indwelling feeding tubes in as little as a week, due to a mixture of microbial colonization and biofilm production [9]. Influencing factors include diet, antimicrobials, stomach acidity, the use of acid-reducing drugs, temperature (inside/outside body), duration of tube implantation, surface characteristics of the tube, and host microbes [3, 5]. The impact of biofilm formation on tube deterioration is similarly multifactorial, with both bacteria and fungi capable of metabolizing tube components [3, 5].

The cases in this series had different underlying diseases, diets, and medications, which likely contributed to differences in individual microbiota and biofilm characteristics.* Candida* sp. and environmental fungi were isolated in this case series.* Candida* sp. are commensal inhabitants of the oral cavity and gastrointestinal tract in humans [10] and are considered part of the oral microbiota in dogs [11]. Simultaneous documentation of the same* Candida* sp. in the mouth, stomach, and G-tube of people suggests that tube colonization generally arises from within the host [10]. Presumably, colonization by* Candida* sp. in the cases reported here arose from commensal yeasts. Definitive conclusions cannot be made in these cases, however, because the lack of anesthesia during tube exchanges precluded collection of concomitant oropharyngeal and gastric cultures [10]. Fungi outside of the host also contribute to tube colonization, as evidenced by isolation of* Curvularia *sp. in this report. Potential sources of exposure include feeding syringes, food-preparation accessories, and human factors. To our knowledge, there are no veterinary guidelines regarding disinfection and/or replacement of feeding tube supplies. Similarly, there is no consensus regarding routine or prophylactic replacement of enteral feeding tubes. One study recommended prophylactic replacement of G-tubes in people if left in place for more than 250 days because of an increased failure rate after that time [2]. All of the cases presented here would fall within the above guidelines.

To our knowledge, the impact of tube colonization on patient outcome is unknown. Stoma-site cellulitis was the primary presenting complaint for one of the cases presented here (case 2). Similarly, one of the investigators (JCW) documented yeast cellulitis causing self-trauma at the stoma site of a long-term indwelling low-profile gastrostomy tube in a dog. The cellulitis was refractory to extensive medical management, but it resolved immediately after replacement of the tube. In one recent retrospective evaluation of E-tubes in cats [12], the top two complications identified were tube dislodgement and stoma-site cellulitis. Unfortunately, data regarding tube colonization were not reported. However, preemptive exchange of colonized E-tubes regardless of tube age seems prudent to decrease the risk of tube dislodgement because stoma-site infection can trigger self-trauma.

Although marked tube colonization was identified in all three E-tubes analyzed in this report, SEM changes consistent with tube deterioration only were noted in one silicone E-tube. The severity of biofilm accumulation prevented accurate structural assessment of the other silicone tube, while the analyzed polyurethane tube had no signs of tube deterioration on SEM analysis. The ideal tube for long-term enteral management is unknown. While mechanical wear will degrade both silicone and polyurethane tubes over time [6, 7], silicone tubes had a greater proportion of intraluminal colonization and altered tube integrity compared to polyurethane tubes in one prospective, randomized-controlled study in people [4]. Conversely, as noted above, some fungi produce hydrolytic enzymes that degrade polyurethane. Additionally, it is important to recognize that certain types of bacteria can metabolize tube components and also play a role in tube degeneration [3, 5]. Given the small number of tubes analyzed, future research is needed to determine if polyurethane tubes are less susceptible to fungal colonization and more resistant to fungal and bacterial degradation or if durability is independent of colonization [6].

Veterinarians should be aware of the association between fungal colonization and tube failure in human medicine. Esophagostomy tubes in dogs and cats with grossly visible brown, black, or creamy blotches should be exchanged. For animals with recurrent issues, investigation into environmental and management factors, such as frequency of disinfection or replacement of feeding syringes, is warranted. In light of how quickly colonization and biofilm develop, consideration should be given to scheduled prophylactic tube exchange. Additional studies which include concomitant oropharyngeal, esophageal, and gastric cultures are needed to clarify whether tube colonization in dogs and cats primarily occurs due to commensal* vs* environmental yeasts and to evaluate factors impacting tube degeneration in veterinary species.
